# Bone Marrow Burkitt Lymphoma in a Child

**DOI:** 10.1155/2020/5606391

**Published:** 2020-09-08

**Authors:** Matija Knežić, Irena Seili Bekafigo, Jelena Roganović, Ita Hadžisejdić, Nives Jonjić

**Affiliations:** ^1^University of Rijeka, Faculty of Medicine, Braće Branchetta 20/1, 51000 Rijeka, Croatia; ^2^Department of Pathology and Cytology, Clinical Hospital Center Rijeka, Cambierieva 17, 51000 Rijeka, Croatia; ^3^Department of Pediatrics, Clinical Hospital Center Rijeka, Istarska ul. 43, 51000 Rijeka, Croatia

## Abstract

Burkitt lymphoma (BL) is a highly aggressive but potentially curable disease as long as adequately treated within due time. BL may occur primarily and exclusively in the bone marrow as a form of peripheral and extranodal disease. BL cases with isolated bone marrow involvement are challenging in regard to a prompt diagnostic process. We report a case of a sporadic extranodal subtype of isolated bone marrow BL in an 11-year-old boy. Bone marrow aspiration and biopsy, flow cytometry, and immunohistochemistry along with cytogenetics are compulsory in order to achieve the adequate diagnosis.

## 1. Introduction

BL is a highly aggressive B-cell NHL characterized by an extremely short doubling time of neoplastic cells and extranodal localization [1]. It constitutes less than 1% of NHL in adults but accounts for 30% of pediatric lymphomas [2]. BL cells are medium-sized, with round nuclei, having multiple basophilic, medium-sized nucleoli and basophilic cytoplasm with vacuoles. BL has characteristic diffuse and monotonous growth pattern with a “starry sky” appearance due to numerous macrophages. BL includes three clinical variants: endemic, sporadic, and immunodeficiency-associated (most commonly in HIV-positive patients) [1]. Bone marrow involvement is common in BL. However, BL exclusively involving the bone marrow without clinical or radiographic evidence of a tumor mass elsewhere is rare. In the past, it has been defined as a pure Burkitt leukemia and was considered as a variant of BL in the WHO classification [1].

We report a case of a rare extranodal presentation of BL, with only bone marrow involvement in an 11-year-old boy with nonspecific clinical findings posing a diagnostic challenge. Early recognition is essential for establishing accurate diagnosis and a prompt tailored treatment.

## 2. Case Report

A previously healthy 11-year-old boy presented to the local hospital with a two-week history of a low-grade fever following an upper respiratory tract infection, intermittent left ankle pain, and fatigue and reduced appetite. Due to mild thrombocytopenia (platelet count 124 × 10^9^/L) and elevated lactate dehydrogenase (1.332 U/L), the patient was transferred to the Tertiary Clinic for Pediatrics for further evaluation. On admission, his condition was good, with palpable nontender cervical nodes (<1.5 cm in diameter) and liver 1 cm below the right costal margin. The initial complete blood count showed hemoglobin 12.1 g/dL, platelet count 117 × 10^3^/*μ*L, and white blood cells 9.7 × 10^9^/L with 5% of blasts in the peripheral blood smear. Sedimentation rate (65 mm/h), serum uric acid (730 *μ*mol/L), and lactate dehydrogenase (1.715 U/L) were elevated. Results of analysis of renal functions and urine were within normal limits.

Flow cytometry of bone marrow aspirate showed positivity for CD10 (19%), CD19 (25%), CD19+kappa+ (3.5%), CD19+lamba+ (22%), and CD20 (24%), suggesting mature B-cell lymphoproliferative disease. Peripheral blood smear showed <5% of atypical blast cells.

**Figure 1:**
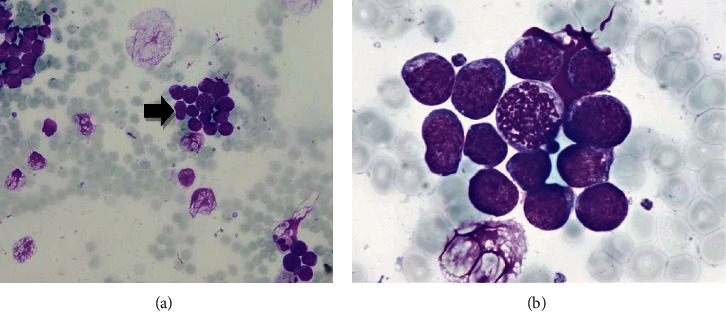
Bone marrow aspirate. Numerous blast cells (arrow) with scant, basophilic cytoplasm containing occasional vacuoles are shown (May-Grünwald-Giemsa: (a) 200x and (b) 1000x).

**Figure 2:**
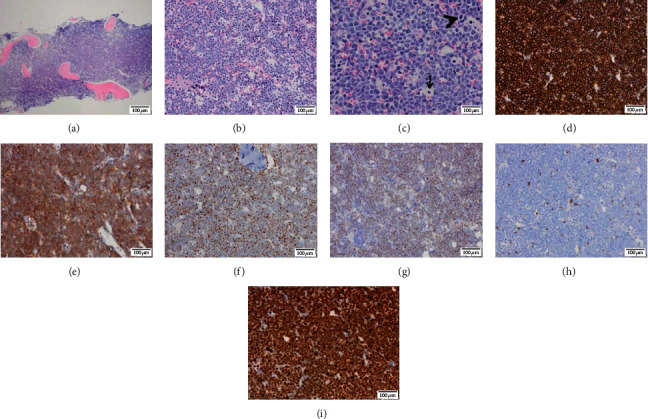
Photographs of bone marrow biopsy with primary Burkitt lymphoma. Low magnification showed a (a) hypercellular bone marrow with (b) monotonous infiltration of medium-sized lymphocytes that displayed (c) many mitotic (arrowhead) and apoptotic figures (arrow). Immunohistochemical analysis of lymphoma cells showed positivity for (d) CD20, (e) CD10, (f) Bcl-6, and (g) c-myc, while (h) Bcl-2 was negative. (i) Ki67 proliferative rate of tumor cells was nearly 100%.

Cytologically, 32% of atypical blasts were found in a bone marrow aspirate. Bone marrow smears were highly cellular, with numerous atypical blasts with a scant, basophilic cytoplasm, occasional small vacuoles, and prominent mitotic figures (Figures 1(a) and 1(b)). Bone marrow biopsy was hypercellular, with almost 100% cellularity due to diffuse monotonous infiltration by medium-sized lymphatic cells. The tumor lymphatic cells had a high proliferation rate, with many mitotic figures and numerous apoptosis. A starry sky pattern with numerous tangible body macrophages was present (Figures 2(a)–2(c)). Immunohistochemically, tumor cells showed positivity for CD20, Bcl-6, CD10, and c-myc, while they were negative for Bcl-2, CD34, TdT, CD3, and CD7. Ki67 was positive in nearly 100% of tumor cells (Figures 2(d)–2(i)).

Bone marrow cytogenetic analysis showed normal chromosome count with *C-MYC* rearrangement and t(8; 14)(q24; q32). Translocation has been confirmed by the fluorescent in situ hybridization (FISH) as well. The lymphatic tumor cells were not found in the cerebrospinal fluid. Further, the whole-body positron emission tomography-computed tomography (PET-CT) for accurate staging was performed. PET-CT demonstrated an increased diffuse (18) F fluorodeoxyglucose bone marrow uptake, without nodal or other extranodal involvement.

The patient underwent chemotherapy according to the B-NHL BFM 04 protocol for stage IV/B-AL (R4 group), which included a prephase and six courses of chemotherapy based on dexamethasone, high-dose methotrexate, ifosfamide, cyclophosphamide, high-dose cytarabine, etoposide, doxorubicin, vincristine, vindesine, and an intrathecal triple-drug therapy (methotrexate/cytarabine/prednisone). The targeted therapy with rituximab was integrated in the treatment from the second course, five doses in total. The complete remission was achieved after one course of chemotherapy. The patient experienced severe side effects including myelosuppression, febrile neutropenia, periapical periodontitis, mucositis, oral and intestinal candidiasis, secondary hypogammaglobulinemia, peripheral neuropathy, and weight loss. The side effects required aggressive supportive treatment with neither delay nor dose reduction of immunochemotherapy. The 11-year boy has been in continuous complete remission for 14 months and is followed up regularly.

## 3. Discussion

Secondary marrow involvement by NHL is relatively common, but lymphomas involving exclusively the bone marrow are very rare [3, 4]. The proposed diagnostic criteria defining primary bone marrow lymphoma are as follows: (1) isolated bone marrow infiltration of lymphoma cells regardless of peripheral blood involvement; (2) no evidence of lymph node, spleen, liver, or other extra bone marrow involvement on physical examination or imaging studies; (3) absence of localized bone tumors; (4) no evidence of bone trabecule destruction in the bone marrow biopsy; and (5) exclusion of leukemia/lymphoma cases that are considered to involve primarily the bone marrow including chronic lymphocytic leukemia/small lymphocytic lymphoma, prolymphocytic leukemia, lymphoplasmacytic lymphoma, mantle cell lymphoma, splenic marginal zone lymphoma, hairy cell lymphoma, BL, and acute lymphoblastic leukemia [5, 6]. Our case fulfills defining criteria 1-4 but not exclusion criterion 5. Most of the reported cases of primary bone marrow lymphoma are diffuse large B-cell lymphomas (DLBCL), but follicular lymphoma, peripheral T-cell lymphoma, and large B-cell lymphoma have also been described [6–11].

In the present case, PET-CT demonstrated an increased diffuse (18) F fluorodeoxyglucose bone marrow uptake, without nodal or other extranodal involvement. Bone marrow examination was crucial to establish the diagnosis. The classification of bone marrow NHL subtypes can be difficult without a solid tumor mass or lymph node specimen. A combination of morphological, immunophenotypic, and genetic findings is helpful for an accurate, definitive diagnosis. In this case, the flow cytometry and immunohistochemical analysis confirmed the peripheral B lymphatic cells and excluded the precursor cells of B lymphoblastic lymphoma.

Common initial clinical presentations of primary bone marrow NHL are nonspecific and include fever mimicking infection, fatigue, anorexia, and weight loss, as presented in the current case. The majority of previously reported cases of primary bone marrow NHL have included bilineage cytopenia or pancytopenia at diagnosis due to bone marrow displacement and autoimmune destruction [12]. Our patient only had a mild thrombocytopenia, whereas in most described cases a severe form of thrombocytopenia with anemia has been shown [13]. In addition, a hypercalcemia and hemophagocytic lymphohistiocytosis can also be a part of clinical presentation in patients with bone marrow lymphoma [11, 14].

The mainstay of the treatment is multiagent immunochemotherapy regimen. The 5-year overall survival of patients with primary bone marrow BL is superior to those with BL and marrow involvement who are similarly treated [15]. In this case, the multiagent chemotherapy treatment with the addition of rituximab was successful, and the patient has been in continuous remission for 14 months.

In conclusion, pediatric BL is a curable disease, with intensive immunochemotherapy. Early clinical findings are nonspecific but require a high index of suspicion. Accurate diagnosis using bone marrow aspiration and biopsy, flow cytometry, immunohistochemistry, and cytogenetics is crucial and mandatory, as well as prompt treatment.
